# cellCounts: an R function for quantifying 10x Chromium single-cell RNA sequencing data

**DOI:** 10.1093/bioinformatics/btad439

**Published:** 2023-07-18

**Authors:** Yang Liao, Dinesh Raghu, Bhupinder Pal, Lisa A Mielke, Wei Shi

**Affiliations:** Olivia Newton-John Cancer Research Institute, Heidelberg, Victoria 3084, Australia; School of Cancer Medicine, La Trobe University, Bundoora, Victoria 3086, Australia; Olivia Newton-John Cancer Research Institute, Heidelberg, Victoria 3084, Australia; School of Cancer Medicine, La Trobe University, Bundoora, Victoria 3086, Australia; Olivia Newton-John Cancer Research Institute, Heidelberg, Victoria 3084, Australia; School of Cancer Medicine, La Trobe University, Bundoora, Victoria 3086, Australia; Olivia Newton-John Cancer Research Institute, Heidelberg, Victoria 3084, Australia; School of Cancer Medicine, La Trobe University, Bundoora, Victoria 3086, Australia; Olivia Newton-John Cancer Research Institute, Heidelberg, Victoria 3084, Australia; School of Cancer Medicine, La Trobe University, Bundoora, Victoria 3086, Australia

## Abstract

**Summary:**

The 10x Genomics Chromium single-cell RNA sequencing technology is a powerful gene expression profiling platform, which is capable of profiling expression of thousands of genes in tens of thousands of cells simultaneously. This platform can produce hundreds of million reads in a single experiment, making it a very challenging task to quantify expression of genes in individual cells due to the massive data volume. Here, we present *cellCounts*, a new tool for efficient and accurate quantification of Chromium data. *cellCounts* employs the seed-and-vote strategy to align reads to a reference genome, collapses reads to Unique Molecular Identifiers (UMIs) and then assigns UMIs to genes based on the *featureCounts* program. Using both simulation and real datasets for evaluation, *cellCounts* was found to compare favourably to *cellRanger* and *STARsolo*. *cellCounts* is implemented in R, making it easily integrated with other R programs for analysing Chromium data.

**Availability and implementation:**

*cellCounts* was implemented as a function in R package *Rsubread* that can be downloaded from http://bioconductor.org/packages/release/bioc/html/Rsubread.html. Data and analysis code used in this study can be freely accessed via La Trobe University’s Institutional Repository at https://doi.org/10.26181/21588276.

## 1 Introduction

The advent of single-cell RNA sequencing (scRNA-seq) technologies has fundamentally transformed biomedical research landscape. scRNA-seq data need to be quantified before downstream analyses can be performed, such as cell clustering, cell type identification, and differential gene expression analysis. Quantifying scRNA-seq data are more complex than quantifying bulk RNA-seq data because in addition to determining the origin of sequence reads, quantification of scRNA-seq data also requires identification of cells and Unique Molecular Identifiers (UMIs). Furthermore, scRNA-seq quantification is more computing intensive due to much larger number of reads generated for a sample. The 10x Genomics Chromium scRNA-seq technology is currently the dominant scRNA-seq platform.

There are two main strategies developed for quantifying data generated by this platform. The first strategy aligns reads to a reference genome and then assigns reads and UMIs to genes in each cell. Examples of methods adopting this strategy include *CellRanger* (Zheng *et al.* 2017) and *STARsolo* (Kaminow *et al.* 2022). The second strategy uses an alignment-free approach to find transcripts compatible with read/UMI sequences and then assign UMIs to transcripts based on a probabilistic model. Representative methods using this strategy include *kallisto|bustools* (Melsted *et al.* 2021) and *alevin-fry* (He *et al.* 2022). In this study, we focus on the alignment-based strategy for quantifying Chromium data.

We present a new Chromium quantification tool called *cellCounts*. *cellCounts* was developed based on the seed-and-vote read mapping paradigm (Liao *et al.* 2013, Liao *et al.* 2019) and *featureCounts* read assignment algorithm (Liao *et al.* 2014, Liao *et al.* 2019). *cellCounts* was implemented as an R function as part of the Bioconductor/R package Rsubread, allowing Chromium quantification to be carried out within the user-friendly R environment. *cellCounts* can work seamlessly with many popular Chromium data analysis R packages to create an R pipeline for a complete analysis of a Chromium dataset. With *cellCounts*, scRNA-seq quantification can be carried out with a single function call (after an index was generated for a reference genome which is a one-off operation). Using both simulation data and real datasets, we demonstrate that the speed and accuracy of *cellCounts* are comparable to, or better than, those of *cellRanger* and *STARsolo*, popular programs developed for quantifying scRNA-seq data.

## 2 Methods

### 2.1 *cellCounts design*

*cellCounts* starts with processing raw reads generated by a sequencer and finishes with outputting UMI counts for each gene in each cell. *cellCounts* is able to take both BCL and FASTQ format reads as input. When input format is BCL, *cellCounts* directly processes reads from the raw data files instead of converting them into FASTQ reads before processing. This avoids the costs of format conversion and disk operations involved in saving and retrieving FASTQ reads. This direct processing of BCL reads could save >20% of *cellCounts’*s running time. It also simplifies the analysis as users do not need to convert BCL reads to FASTQ reads which is required by other tools such as *CellRanger*.

*cellCounts* adapted the seed-and-vote aligner *Subread* for mapping Chromium reads. *cellCounts* performs more sensitive read mapping than *Subread*, by using more seeds (15 seeds) to discover candidate mapping locations and by applying a more relaxed voting threshold for calling mapping locations (only requiring a minimum of one vote). This highly sensitive read mapping enables more UMIs to be detected from the scRNA-seq data. *cellCounts* considers both numbers of matched bases and mismatched bases in each alignment to find the best mapping location. This ensures a high mapping accuracy is achieved. If a read maps to more than one location, the location overlapping a known gene is preferred.

Mapped reads will be assigned to genes in each cell using the *featureCounts* algorithm. Within each gene, assigned reads that share the same UMI tag (allowing one base mismatch) will be reduced to one UMI. If the same UMI appears in more than one gene, it will be assigned to the gene that has the highest number of reads carrying this UMI tag. *cellCounts* supports barcode correction and whitelisting. It utilizes a barcode whitelist, which is a list of barcode sequences downloaded from 10x Genomics website that are used by Chromium assay kit, to detect valid barcodes in a Chromium dataset. When matching cell barcodes observed from the data against the barcode whitelist, *cellCounts* allows for one base mismatch to account for sequencing errors.

After obtaining UMI counts for each gene in each cell, *cellCounts* will call valid cells using the same algorithm as implemented in *CellRanger* (version 3.0). Briefly, this algorithm first uses a bootstrap sampling procedure to determine a total-UMI-count cut-off for calling high-confidence cell barcodes. It then builds an expression profile for ambient RNAs based on expression levels of genes in those barcodes that have a very low total UMI count. Lastly, *CellRanger* will rescue the cell barcodes that fail to meet the total-UMI-count cut-off but have a gene expression profile significantly different from the ambient RNA profile. *cellCounts* reports both high-confidence cells and rescued cells in the UMI count matrix it generates.

### 2.2 Simulation

A simulation dataset was generated for this evaluation, based on the *CellRanger* analysis results of a Chromium dataset including human peripheral blood mononuclear cells (PBMCs) of a healthy female donor. This PBMC dataset was generated by 10x Genomics. The library was prepared using the Chromium Next GEM v3.1 chemistry and sequencing was performed using an Illumina NovaSeq 6000. We downloaded this dataset from 10x Genomics website. We used the quantification statistics observed from the *CellRanger* quantification results to generate the simulation data. Expression of genes in 10, 000 cells was simulated. Number of genes per cell was subject to a Gamma distribution (*k* = 4.5, θ = 550), with an average of 1,930 expressed genes included in each cell. UMIs per gene and reads per UMI were subject to Gamma distribution with parameter settings of *k* = 1, θ = 6 and *k* = 2, θ = 2, respectively. Approximately 50k reads were generated for each cell on average. Each simulation read includes a technical part and a biological part. The technical part contains a sample index sequence, a cell barcode sequence and a UMI sequence. We downloaded technical sequences from 10x Genomics website and used them for this simulation. The biological part of a simulation read includes a genomic sequence extracted from the 3' end of a gene. This mimics the 3' bias of Chromium Single Cell 3′ GEM library. This genomic sequence is located 350 bases from the very end of the gene on average with a standard deviation of 30 bases. Genomic variants and sequencing errors were introduced to reads using the same approach as described in Liao *et al.* (2019). Briefly, sequencing quality data were obtained from a real Illumina sequencing dataset. Quality scores included in the data were provided to both technical and biological parts of simulation reads. Substitution errors were then introduced to read bases based on quality scores assigned to the bases. More errors were introduced to the 3' end of simulation reads due to sequencing bias of Illumina technology. Biological variants were only introduced to the biological part of simulation reads. These variants included single nucleotide polymorphisms and short indels, which were introduced at the rates of 0.0009 and 0.0001, respectively.

### 2.3 Software execution

*CellRanger* (version 6.0.1), *STARsolo* (version 2.7.10a) and *cellCounts* (version 2.14.2) were executed on a supercomputer with 48 Intel Xeon 3.30 GHz CPU cores and 768 GB of memory. Each program was specified to run with 10 CPU threads. Note that the *STARsolo* program utilizes two internal threads to decompress read files and distribute reads to other threads, resulting in a total of 12 threads used for quantification. As both *CellRanger* and *cellCounts* output location-sorted BAM files by default, we also instructed *STARsolo* to output location-sorted BAM files unless otherwise stated. The pre-built *CellRanger* reference package provided by 10x Genomics was used for running *CellRanger*. Reference sequences and gene annotation included in the reference package were also provided to *STARsolo* and *cellCounts* for their data quantification.

## 3 Results

### 3.1 Speed

We first assessed the speed of *cellCounts*, *CellRanger* and *STARsolo*. Two in-house mouse datasets, including 245 and 379 million reads, respectively, and a public mouse melanoma dataset including 505 million reads (Gene Expression Omnibus database accession GSM4505965), were used in this evaluation. The in-house datasets were generated using Chromium Next GEM v3.1 chemistry. The smaller in-house dataset includes CD45^+^ cells sorted from stomach, small intestine, caecum and colon. The larger in-house dataset includes TCRβ^+^CD8α^+^ and TCRγδ^+^CD8α^+^ cells sorted from colon. The public dataset includes tumour-infiltrating leukocytes sorted from a melanoma sample using Chromium Next GEM v2 chemistry.

**Figure 1.**
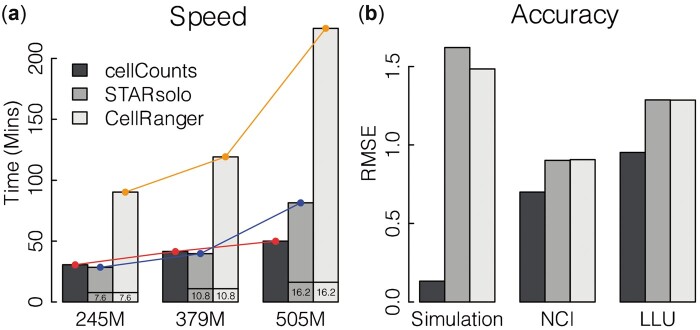
Comparison of speed and accuracy for quantifying 10x Chromium scRNA-seq data. (a) Running time of *cellCounts*, *STARsolo* and *CellRanger* on three real datasets. Number of reads included in each dataset is indicated under each column. These datasets all have a BCL format. Numeric values shown at the bottom of bars for *STARsolo* and *CellRanger* indicate the amount of time spent on converting BCL-format reads to FASTQ-format reads. *cellCounts’*s running time does not include this time because *cellCounts* directly processes BCL-format reads. (b) Root mean square error (RMSE) of gene expression calculated for *cellCounts*, *STARsolo* and *CellRanger* based on ground truth. For each method, UMI counts were converted to log2-cpm values for each gene in each cell and then used for calculating RMSE. An offset of 0.5 was added to UMI counts to avoid log transformation of zero counts. ‘Simulation’ is a simulation dataset. ‘NCI’ and ‘LLU’ are two real datasets generated from sequencing of two human cell lines mixed at known ratios.

Figure 1a shows that both *cellCounts* and *STARsolo* are significantly faster than *CellRanger. cellCounts* is 1.7–3.3 times faster than *CellRanger. cellCounts* demonstrates comparable speed to *STARsolo*, while exhibiting better scalability over large data size, as illustrated by the red line in Fig. 1a. Similar comparison results were observed when the output of BAM files is not needed.

### 3.2 Accuracy

We then compared the quantification accuracy of the three quantification tools. We first generated a simulation dataset for this evaluation, based on the *CellRanger* analysis results of a PBMC dataset generated by using 10x Chromium Next GEM v3.1 chemistry. Quantification statistics obtained from *CellRanger* analysis results were used to set parameters for Gamma distributions used to draw number of genes per cell, number of UMIs per gene and number of reads per UMI. Simulation reads were extracted from 3' end of each gene to mimic the 3' bias of Chromium Single Cell 3′ GEM library. Genomic variants and sequencing errors were also introduced to read sequences. Details of generation of simulation data can be found in Section 2.2.

A UMI count matrix including known expression levels (UMI counts) generated for each gene in each cell in the simulation, was used as the ground truth for assessing quantification accuracy of *CellRanger*, *STARsolo* and *cellCounts*. The ‘Simulation’ column in Fig. 1b shows that *cellCounts* is more accurate (smaller RMSE) than *CellRanger* and *STARsolo*. *cellCounts* successfully recalled all the cells generated in the simulation, whereas *CellRanger* and *STARsolo* failed to call ∼2% of the cells. Cell calling is performed based on gene expression profiles in the cells. The better cell calling result from *cellCounts* should be due to its read mapping and UMI assignment algorithm, which is the main difference between *cellCounts* and the other two tools.

We then used two real 10x Chromium datasets, generated by a recent multicentre scRNA-seq benchmarking study (Chen *et al.* 2021), to compare the quantification accuracy of the three methods. Each dataset contained data generated from a breast cancer cell line sample (sample ‘A’), a normal B lymphocyte cell line sample (sample ‘B’) and a mixture of the two samples. The mix ratio is 95%A:5%B for dataset ‘NCI’ and 90%A:10%B for dataset ‘LLU’. The RMSE quantification error was calculated by comparing expression levels of genes in the mixture sample and sum of expression levels of genes in sample ‘A’ and ‘B’, multiplied by their respective mix ratio. In other words, we examined which quantification method yielded better concordance of gene expression between the *in silico* mixture sample and the actual mixture sample. Using known mix ratios as truth to assess gene expression accuracy has been demonstrated to be a successful strategy for evaluating the performance of RNA-seq quantification methods (SEQC/MAQC-III Consortium 2014). With this strategy, we found that *cellCounts* generated more concordant quantification results than *CellRanger* and *STARsolo* (‘NCI’ and ‘LLU’ columns in Fig. 1b).

In summary, we have developed a new tool called *cellCounts*, specifically designed for quantifying 10x Chromium scRNA-seq data in this study. The performance of *cellCounts* has been evaluated using both simulated and real data, demonstrating its effectiveness. It allows users to perform quantification within the user-friendly R environment and facilitates the creation of an R pipeline for analysing Chromium data, starting from raw reads and leading to final results.

## References

[btad439-B1] Chen W , Zhao Y, Chen X et al A multicenter study benchmarking single-cell RNA sequencing technologies using reference samples. Nat Biotechnol 2021;39:1103–14. 33349700 10.1038/s41587-020-00748-9 PMC11245320

[btad439-B2] He D , Zakeri M, Sarkar H et al Alevin-fry unlocks rapid, accurate and memory-frugal quantification of single-cell RNA-seq data. Nat Methods 2022;19:316–22. 35277707 10.1038/s41592-022-01408-3 PMC8933848

[btad439-B3] Kaminow B, Yunusov D, Dobin A. STARsolo: accurate, fast and versatile mapping/quantification of single-cell and single-nucleus RNA-seq data. *bioRxiv* 2022. 10.1101/2021.05.05.442755.

[btad439-B4] Liao Y , Smyth GK, Shi W et al The subread aligner: fast, accurate and scalable read mapping by seed-and-vote. Nucleic Acids Res 2013;41:e108. 23558742 10.1093/nar/gkt214 PMC3664803

[btad439-B5] Liao Y , Smyth GK, Shi W et al featureCounts: an efficient general purpose program for assigning sequence reads to genomic features. Bioinformatics 2014;30:923–30. 24227677 10.1093/bioinformatics/btt656

[btad439-B6] Liao Y , Smyth GK, Shi W et al The R package Rsubread is easier, faster, cheaper and better for alignment and quantification of RNA sequencing reads. Nucleic Acids Res 2019;47:e47. 30783653 10.1093/nar/gkz114 PMC6486549

[btad439-B7] Melsted P , Booeshaghi AS, Liu L et al Modular, efficient and constant-memory single-cell RNA-seq preprocessing. Nat Biotechnol 2021;39:813–8. 33795888 10.1038/s41587-021-00870-2

[btad439-B8] SEQC/MAQC-III Consortium. A comprehensive assessment of RNA-seq accuracy, reproducibility and information content by the sequencing quality control consortium. Nat Biotechnol 2014;32:903–14. 25150838 10.1038/nbt.2957 PMC4321899

[btad439-B9] Zheng GXY , Terry JM, Belgrader P et al Massively parallel digital transcriptional profiling of single cells. Nat Commun 2017;8:14049. 28091601 10.1038/ncomms14049 PMC5241818

